# Comparison Between the Accuracy of NovApex Apex Locator and Radiographs in Determining Radiographic Apex

**Published:** 2011-05-15

**Authors:** Shahriar Shahi, Saeed Rahimi, Amin Salem Milani, Mohammad Asghari Jafarabadi, Gholam Reza Djoorabbaf Naghsh

**Affiliations:** 1. Department of Endodontics, Dental and periodontal disease research center, Dental School, Tabriz University of Medical Sciences, Tabriz, Iran.; 2. Department of Endodontics, Dental School, Tabriz University of Medical Sciences, Tabriz, Iran.; 3. Department of Statistics and Epidemiology, Health and Nutrition School, Tabriz University of Medical Sciences, Tabriz, Iran.; 4. Dentist, Private Practice, Tabriz, Iran.

**Keywords:** Electronic Apex Locator, NovApex, Radiographic Image Interpretation

## Abstract

**INTRODUCTION:**

Determination of the canal working length with radiographs has many drawbacks. Electronic apex locators have been developed to overcome some of these problems. Recently, a newly designed apex locator called NovApex has been introduced. All the studies conducted to determine the accuracy of NovApex have been carried out in-vitro on extracted teeth. The aim of this in vivo study was to evaluate the accuracy of NovApex compared with traditional radiographic method.

**MATERIALS AND METHODS:**

Twenty-five patients whose lower molars that were candidates for extraction were selected. The teeth were accessed, and the radiographic working length was determined by measuring the length of the initial file 0.5mm short of the radiographic apex. Then, NovApex apex locator was used to measure the electronic working length. Subsequently, the tooth was extracted, and the actual working length was measured by introducing a size #15 K-file into each canal until the file tip was visible at the apex, then 1.5mm was subtracted to attain the working length. Cohen's Kappa was computed for each of the methods versus actual working length as a measure of reliability. The accuracies were compared using Chi-square test.

**RESULTS:**

The accuracy of NovApex apex locator and radiographic method in detecting the apical end point within ±0.5mm was 74.7% and 68%, respectively; this was not significantly different (P<0.001).

**CONCLUSION:**

The NovApex apex locator is useful in detecting the apical end point with the accuracy similar to radiographic method. However, neither technique is fully reliable in detecting the apical end point of the canal.

## INTRODUCTION

Adequate cleaning and disinfection of the root canal system is required for successful treatment. Therefore, accurate detection of apical end point of the canal is a crucial step in endodontic treatment. Sjogren et al. showed that teeth filled within 0 to 2mm from the apex enjoyed 94% success rate, falling to 76% in over-filled teeth and to 68% in under-filled teeth (≥2mm) [1]. Several methods have been used to determine the working length. Radiographic method is the most widely used technique. However, this method gives a two-dimensional image of the roots, and determination of the working length by this method is not always accurate [2].

Furthermore, superimposition of other structures usually makes working length determination difficult [3][4]. Tooth inclination and angulation of the x-ray tube also influence the results [5]. Custer for the first time proposed an electronic method to determine root canal length [6], and later, another researcher invented the first electronic apex locator (EAL) [7]. Since then, different generations of EALs have been developed. The main concern in using EALs is the accuracy of the measurements. The measurements made by early devices were influenced by canal contents, remnants of pulp tissue, bleeding or pus [8][9]. However, new generations claimed to have more accuracy and less sensitivity to canal contents. Recently, a newly designed EAL NovApex (Forum Technologies, Rishon Le-Zion, Israel) has been introduced. This device utilizes voltage differences and operates on the principle that impedance differs between two electrodes, depending on the frequencies used, and also differs greatly on the apical constriction [4]. Since the introduction of NovApex, several studies have been carried out to evaluate its accuracy which showed that the accuracy of NovApex in detecting the apical end point of the canal is more than 80% [4][10][11]. However, all of these studies were carried out in vitro on extracted teeth, and the condition in which the measurements were performed did not completely simulate the clinical situation. The aim of this study was to evaluate the accuracy of the NovApex in vivo and compare it with traditional radiographic method.

## MATERIALS AND METHODS

The study protocol was in accordance with the Declaration of Helsinki, as revised in 2004 [12]. Twenty-five patients whose lower first or second molar with the diagnosis of irreversible pulpitis was candidate for extraction were selected. The diagnosis was confirmed by thermal tests. Written informed consent was obtained from the volunteers. A periapical radiograph with paralleling technique was obtained as the initial radiograph. If there was any sign of previous root canal therapy or canal obliteration, the case would not be included. The tooth was anesthetized with inferior alveolar nerve block injection of 1.8 mL of 2% lidocaine with 1:80,000 epinephrine. After access cavity preparation and tooth isolation, the coronal pulp was removed using a spoon excavator. If the pulp looked non-vital or partially necrotic, again the case was not included. The canals were negotiated with a size #15 K-file (Maillefer, Ballagius, Switzerland). The radiographic working length (RWL) was estimated from the initial radiograph 0.5mm short of the radiographic apex and confirmed with another radiograph with a proper initial file in place. Then, another operator measured the electronic working length (EWL) using NovApex apex locator (Forum Technologies, Rishon Le-Zion, Israel). For this purpose, the access cavity was dried with cotton pellets. Then, the EWL was measured using a size 20 Flexofile (Maillefer, Switzerland) attached to the file holder of the apex locator. The file was advanced apically until the signal indicated the apical foramen. The file was retracted until the digital display read 0.5mm. The measurements were made twice, and the mean was recorded as the EWL of the canal. Subsequently, the tooth was extracted and cleaned with a periodontal curette under running water. A size #15 K-file (Maillefer, Ballagius, Switzerland) was inserted into each canal until the file tip was visible at the apex under stereomicroscope (Carl Zeiss, CITY Germany) at ×20 magnification. One half millimeter was subtracted from this length and recorded as the actual working length (AWL).

### Statistical analysis

Statistical analysis was performed by SPSS for windows (SPSS Inc, Chicago, IL). The significance level was set at 0.05. The accuracy of the NovApex apex locator and radiographic method was calculated within ±0.5mm. Chi-square test was used to compare the accuracy of the two methods. Cohen's Kappa was computed for each of the methods versus actual working length as a measure of reliability (agreement). Values over 0.75 confirm a fair reliability of the results

## RESULTS

The accuracy of NovApex apex locator and radiographic method within ± 0.5mm was 74.7% and 68%, respectively which was not significantly different (χ^2^=.815, df=1, P=0.367) (Table 1).

**Table 1:** Accuracy of electronic and radiographic methods vis-a-vis actual working length method

**Method **	**Accurate (n)**	**Accurate (%)**
**EWL^a^ vs. AWL^b^**	56/75	(74.7%)
**RWL^c^ vs. AWL**	51/75	(68%)

^a^ Electronic working length

^b^ Actual working length

^c^ Radiographic working length (χ^2^=0.815, df=1, p=0.367. Cohen's Kappa = .773, P<0.001)

## DISCUSSION

The determination of working length is a key factor for successful endodontic treatment. The electronic working length measurement has become increasingly popular because it eliminates many of the problems associated with traditional radiographic methods [13]. Since the introduction of electronic apex locators, various studies have evaluated the accuracy of these devices [13]. These studies analyze a range of brands of apex locator and the methods to measure the accuracy [2][10][11][13][14][15][16][17][18][19][20][21][22][23]. Most of these studies were carried out in-vitro on extracted teeth [2][10][11][14][16][17][18][19][20][21][22][23][24][25][26][27][28].The present study was performed in an in-vivo condition to increase clinical relevancy of the results. This study showed that accuracy of NovApex apex locator within ±0.5mm was 74.7%. We used a tolerance limit of ±0.5 as it is considered to be the strictest acceptable range [29]. However, some older studies rely on a more flexible clinical range of ±1.0mm [30]. An ex vivo study carried out by D’Assuncao et al. on extracted teeth showed that the accuracy of NovApex in determining the apical foramen within ±0.5mm was 82.1% [4]. The apical end point used in that study was the apical foramen. However, in our study, 0.5mm short of the apical foramen was considered as the apical end point, and the electronic measurements were performed in-vivo. In another in-vitro study, the electronic measurements obtained with NovApex in retreatment cases showed an accuracy of 85% (within ±0.5mm of the apical constriction) [11]. Although the accuracy value obtained in our study is less than above mentioned in vitro studies, it has relatively high accuracy similar to the radiographic method (Table 1). One study showed that NovApex is able to detect the position of the horizontal root fracture in extracted teeth with accuracy of 70% within ±0.5mm of the fracture line [10]. Because our study was performed in a real clinical situation, the clinical validity of the results is greater than the above mentioned studies. Previous studies showed no statistically significant difference between NovApex and Root ZX [4][10][11]. It is recommended that this conclusion also be confirmed in real clinical situations.

The results of our study demonstrated that the accuracy of NovApex apex locator in detecting the apical end point of the canal is more than traditional radiographic technique (74.7% vs. 68.0%); although the difference was not statistically significant (P>0.05). This result is in accordance with other studies that compared the accuracy of electronic apex locators with radiographic technique [13][31].

## CONCLUSION

It can be concluded that NovApex is useful for measuring the working length with accuracy similar to traditional radiographic methods. However, neither technique has 100% reliability in detecting the apical end point of canals.

## References

[1] Sjogren U, Hagglund B, Sundqvist G, Wing K (1990). Factors affecting the long-term results of endodontic treatment.. J Endod.

[2] De Moor RJ, Hommez GM, Martens LC, De Boever JG (1999). Accuracy of four electronic apex locators: an in vitro evaluation.. Endod Dent Traumatol.

[3] Tamse A, Kaffe I, Fishel D (1980). Zygomatic arch interference with correct radiographic diagnosis in maxillary molar endodontics.. Oral Surg Oral Med Oral Pathol.

[4] D'Assuncao FL, de Albuquerque DS, de Queiroz Ferreira LC (2006). The ability of two apex locators to locate the apical foramen: an in vitro study.. J Endod.

[5] Palmer MJ, Weine FS, Healey HJ (1971). Position of the apical foramen in relation to endodontic therapy.. J Can Dent Assoc (Tor).

[6] Custer LE (1918). Exact method of locating the apical foramen.. J Natl Dent Assoc.

[7] Sunada I (1962). New method for measuring the length of the root canal.. J Dent Res.

[8] Trope M, Rabie G, Tronstad L (1985). Accuracy of an electronic apex locator under controlled clinical conditions.. Endod Dent Traumatol.

[9] Oishi A, Yoshioka T, Kobayashi C, Suda H (2002). Electronic detection of root canal constrictions.. J Endod.

[10] Goldberg F, Frajlich S, Kuttler S, Manzur E, Briseno-Marroquin B (2008). The evaluation of four electronic apex locators in teeth with simulated horizontal oblique root fractures.. J Endod.

[11] Goldberg F, Marroquin BB, Frajlich S, Dreyer C (2005). In vitro evaluation of the ability of three apex locators to determine the working length during retreatment.. J Endod.

[12] Finish medical association. (2010). WMA Declaration of Helsinki. Helsinki.

[13] Shanmugaraj M, Nivedha R, Mathan R, Balagopal S (2007). Evaluation of working length determination methods: an in vivo/ex vivo study.. Indian J Dent Res.

[14] al Kadi H, Sykes LM, Vally Z (2006). Accuracy of the Raypex-4 and Propex apex locators in detecting horizontal and vertical root fractures: an in vitro study. Sadj.

[15] Busch LR, Chiat LR, Goldstein LG, Held SA, Rosenberg PA (1976). Determination of the accuracy of the Sono-Explorer for establishing endodontic measurement control.. J Endod.

[16] Carneiro E, Bramante CM, Picoli F, Letra A, da Silva Neto UX, Menezes R (2006). Accuracy of root length determination using Tri Auto ZX and ProTaper instruments: an in vitro study.. J Endod.

[17] D'Assuncao FL, de Albuquerque DS, Salazar-Silva JR, de Queiroz Ferreira LC, Bezerra PM (2007). The accuracy of root canal measurements using the Mini Apex Locator and Root ZX-II: an evaluation in vitro.. Oral Surg Oral Med Oral Pathol Oral Radiol Endod.

[18] Ebrahim AK, Wadachi R, Suda H (2007). An in vitro evaluation of the accuracy of Dentaport ZX apex locator in enlarged root canals.. Aust Dent J.

[19] Javidi M, Moradi S, Rashed R, Raziee L (2009). In vitro comparative study of conventional radiography and Root ZX apex locator in determining root canal working length.. N Y State Dent J.

[20] Jenkins JA, Walker WA, 3rd, Schindler WG, Flores CM (2001). An in vitro evaluation of the accuracy of the root ZX in the presence of various irrigants.. J Endod.

[21] McDonald NJ, Hovland EJ (1990). An evaluation of the Apex Locator Endocater.. J Endod.

[22] Nekoofar MH, Sadeghi K, Sadighi Akha E, Namazikhah MS (2002). The accuracy of the Neosono Ultima EZ apex locator using files of different alloys: an in vitro study.. J Calif Dent Assoc.

[23] Tinaz AC, Maden M, Aydin C, Turkoz E (2002). The accuracy of three different electronic root canal measuring devices: an in vitro evaluation.. J Oral Sci.

[24] Kaufman AY, Keila S, Yoshpe M (2002). Accuracy of a new apex locator: an in vitro study.. Int Endod J.

[25] Guise GM, Goodell GG, Imamura GM (2010). In vitro comparison of three electronic apex locators.. J Endod.

[26] Stoll R, Urban-Klein B, Roggendorf MJ, Jablonski-Momeni A, Strauch K, Frankenberger R (2010). Effectiveness of four electronic apex locators to determine distance from the apical foramen.. Int Endod J.

[27] Jung IY, Yoon BH, Lee SJ (2011). Comparison of the reliability of "0.5" and "APEX" mark measurements in two frequency-based electronic apex locators.. J Endod.

[28] Real DG, Davidowicz H, Moura-Netto C, Zenkner Cde L, Pagliarin CM, Barletta FB, de Moura AA (2011). Accuracy of working length determination using 3 electronic apex locators and direct digital radiography.. Oral Surg Oral Med Oral Pathol Oral Radiol Endod.

[29] Haffner C, Folwaczny M, Galler K, Hickel R (2005). Accuracy of electronic apex locators in comparison to actual length-an in vivo study.. J Dent.

[30] Keller ME, Brown CE Jr, Newton CW (1991). A clinical evaluation of the Endocater-an electronic apex locator.. J Endod.

[31] Pratten DH, McDonald NJ (1996). Comparison of radiographic and electronic working lengths.. J Endod.

